# B7-H3 membranous expression correlates with histological grading in a two-center cohort of 133 pre-treatment bone and soft tissue sarcoma samples

**DOI:** 10.1186/s12885-026-16255-0

**Published:** 2026-06-06

**Authors:** Linus D. Kloker, Robert Rottscholl, Jonas S. Heitmann, Peter Bronsert, David Braig, Katrin Benzler, Lars Zender, Christoph K. W. Deinzer

**Affiliations:** 1 Department of Internal Medicine VIII - Medical Oncology and Pneumology, Medical University Hospital, Tübingen, Germany; 2 Center for Soft Tissue Sarcomas, GIST and Bone Tumors (ZWS) of the University Hospital Tübingen and the Comprehensive Cancer Center (CCC) Tübingen-Stuttgart, Tübingen, Germany; 3 DFG Cluster of Excellence 2180 ‘Image-Guided and Functional Instructed Tumor Therapy’ (iFIT), University of Tübingen, Tübingen, Germany; 4 Department of Pathology, University Hospital Tübingen, Tübingen, Germany; 5 Department of Internal Medicine, Clinical Collaboration Unit Translational Immunology, University Hospital Tübingen, Tübingen, Germany; 6 Institute for Surgical Pathology, Medical Center, Faculty of Medicine, University of Freiburg, Freiburg, Germany; 7 Sarcoma Center of the Comprehensive Cancer Center Freiburg (CCCF), Freiburg, Germany; 8 Department of Plastic and Hand Surgery, Faculty of Medicine, Medical Center - University of Freiburg, University of Freiburg, Freiburg, Germany

**Keywords:** B7-H3 (CD276), Soft tissue sarcoma, Bone sarcoma, Gastrointestinal stromal tumor (GIST), B7-H3 membranous expression, Histological grading

## Abstract

**Background:**

B7-H3 targeted therapies are an upcoming promising treatment option for a variety of solid tumors. High levels of B7-H3 expression have only been reported for a few sarcoma subtypes in previous studies, but a systematic analysis in an untreated sarcoma cohort and across many different sarcoma subtypes has not yet been conducted.

**Methods:**

One hundred thirty-three sarcoma samples of previously untreated patients from different sarcoma entities including soft tissue sarcoma (STS, *n* = 103) and bone sarcoma (BS, *n* = 30) were collected from two sarcoma centers in South-West Germany. Samples were centrally immunohistochemically stained and assessed for membranous B7-H3 expression. For further analysis of B7-H3 expression, H-Scores were calculated. Baseline characteristics such as sex, age, histology, grading, staging as well as outcome data like treatments, event-free and overall survival were collected.

**Results:**

B7-H3 was found to be broadly expressed (97%) across all tested sarcoma subtypes, with only four samples (3%) without B7-H3 expression. 45% of all samples had low (H-Score 1-100), 33% intermediate (H-Score 101–200) and 19% high (H-Score 201–300) membranous B7-H3 expression. Highest expression was found in pleomorphic liposarcoma (median 202.5), osteosarcoma (median 175) and angiosarcoma (median 167.5), whereas expression was lowest in myxoid and well differentiated liposarcoma (medians 44.5 & 62.5) and chondrosarcoma (median 60). H-Scores were found to significantly correlate positive with histological grading irrespective of histological sarcoma subtype. Pre-treatment B7-H3 H-Scores did predict event-free but not overall survival.

**Conclusions:**

B7-H3 is a promising treatment target in sarcomas as it is highly expressed particular in high grade bone and soft tissue sarcomas, such as pleomorphic liposarcomas and osteosarcomas.

**Supplementary Information:**

The online version contains supplementary material available at 10.1186/s12885-026-16255-0.

## Introduction

Metastatic soft tissue sarcomas (STS) and bone sarcomas (BS) are a heterogenous group of tumors and are still associated with adverse survival prognosis and a lack of promising treatment options besides conventional chemotherapies in the metastatic situation. At the moment, numerous novel systemic therapies for locally advanced or metastatic sarcomas are under investigation in clinical trials [1–5].

The transmembrane protein B7-H3 has been described as a potential therapeutic targeted in a broad variety of tumor entities. It has immunoregulatory functions by suppression of T-cell and NK-cell activation as well as macrophage polarization towards an M2 phenotype [6, 7]. While its expression is downregulated at the transcriptional level and suppressed post transcriptionally by microRNAs (miRNAs) in nonmalignant tissue, B7-H3 has been shown to be overexpressed and associated with increased invasiveness and metastatic potential in a broad range of tumor entities [8, 9]. In malignant tumors, it was shown to directly promote proliferation, glycolysis, tumor cell migration and invasion [9–14]. A pan-cancer analyses using transcriptomic data of over 156,000 samples showed the second and third highest expression of B7-H3 in STS and BS across 50 distinct tumor types [15]. In this study, 52.5% of all sarcomas were classified as B7-H3 high expressors, showing expression within the top quartile of the pan-cancer cohort. For BS, high B7-H3 expression was correlated with poorer OS.

So far, there is no B7-H3 targeted drug with an FDA (Food and Drug Administration) or EMA (European Medicines Agency) approval, but numerous B7-H3 directed therapies, including monoclonal antibodies (mAbs), antibody drug conjugates (ADCs), bispecific antibodies (bsAbs), radioimmunotherapy and CAR-T cells are under investigation in clinical trials [16–19].

To date, only very limited evidence exists on the differential expression scores of B7-H3 across various sarcoma subtypes and histological grades. Also, reporting of B7-H3 expression ranges from transcriptomic data (transcripts) to dichotomous (+/-), hierarchical (1+, 2+, 3+) or continuous (H-Scores) IHC scoring [15, 19–22]. In this study, primary sarcoma samples from all histologic subtypes were evaluated by immunohistochemistry for membranous B7-H3 expression. The objectives of the study were to investigate the association between B7-H3 expression in untreated sarcomas and clinical factors such as histological subtype, tumor size, nodal status, metastases or histological grading. Further, influence of B7-H3 expression on event-free survival (EFS) as well as on overall survival (OS) was assessed.

## Methods

### Patient enrollment

Patients diagnosed with soft tissue sarcomas (STS) including well-differentiated liposarcoma (WDLPS), dedifferentiated liposarcoma (DDLPS), myxoid liposarcoma (MLPS), pleomorphic liposarcoma (PLPS), leiomyosarcoma (LMS), alveolar rhabdomyosarcoma (ARMS), embryonal rhabdomyosarcoma (ERMS), synovial sarcoma (SS), angiosarcoma (AS) and undifferentiated pleomorphic sarcoma (UPS) as well as patients diagnosed with bone sarcomas (BS) including osteosarcoma (OSS), chondrosarcoma (CS) and Ewing sarcoma (ES) between 2012 and 2024 at the German sarcoma centers of the University Hospital of Tübingen and the University Hospital of Freiburg were enrolled. Only patients with pre-treatment samples obtained from the primary tumor were eligible. Informed consent was obtained from all patients, and the study was conducted according to the declaration of Helsinki. The study was approved by the local Institutional Review Board of the University of Tübingen (Ethics Committee of the Faculty of Medicine of the University of Tübingen, reference no. 241/2024BO2) and the University of Freiburg (Ethics Committee of the Faculty of Medicine of the University of Freiburg, reference no. 21-1735). Clinical trial number: not applicable.

### B7-H3 staining

Immunostaining was performed centralized at the Institute of Pathology of the University Hospital of Tübingen on an automated immunostainer (Ventana Benchmark XT, Ventana, Tucson, AZ, USA). A rabbit monoclonal antibody directed against B7-H3 was used (1:75, BIO SB, Santa Barbara, CA, USA, Clone B7-H3) and validated in testis, tonsil, ovarian carcinoma and carcinoma of the cervix, as recommended by the manufacturer. Intra- and peritumoral blood vessels showing high and stable B7-H3 expression served as internal positive control. Heat induced antigen retrieval was done for 64 min at 95 °C in TRIS-EDTA buffer. The primary antibody was incubated for 32 min at 37 °C. Visualization was done using the OptiView DAB Detection Kit (Roche Diagnostics, Rotkreuz, Switzerland).

### Evaluation and H-Score

**Fig. 1:**
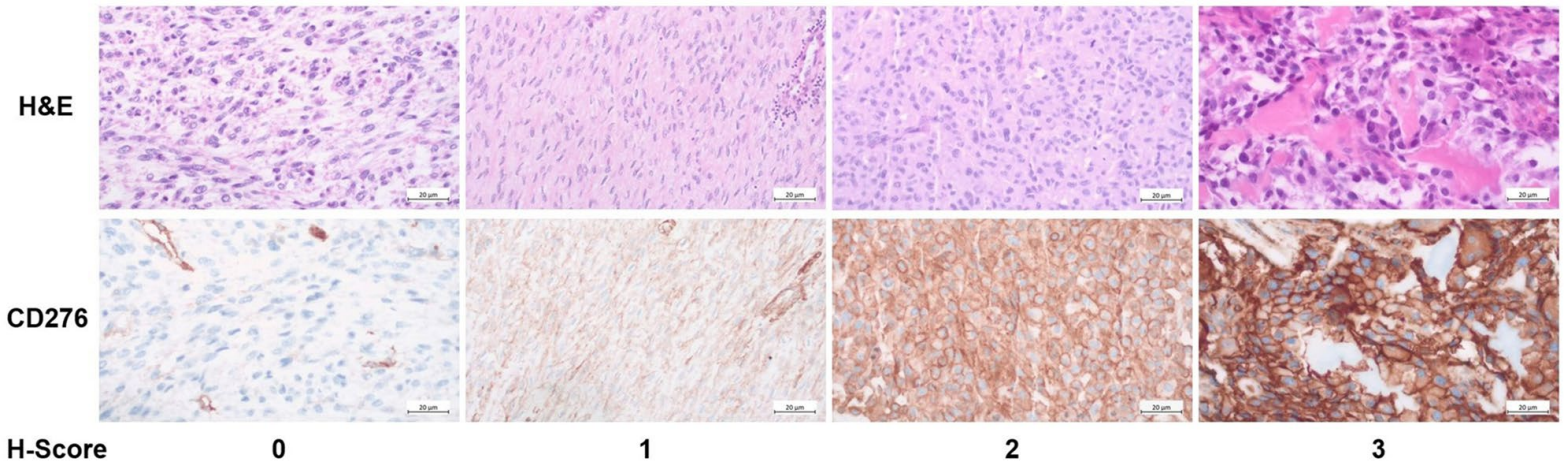
Representative immunohistochemical stains for B7-H3 scoring. Images of each immunohistochemical membranous staining intensity (0–3) for B7-H3. With staining intensity zero, only tumor vasculature is positive for B7-H3

The tumor containing area was verified on freshly cut H&E slides. The immunohistochemical staining of tumor cell membranes was scored by a board-certified pathologist as previously published [23]. Briefly, the percentage of tumor cells staining with low, moderate and high membranous intensities was individually scored at 10x magnification and weighed 0x, 1x, 2x and 3x according to the particular staining intensity. The sum of these scores were calculated to the H-Score resulting in values ranging from 0 to 300. Representative images for staining intensities are shown in Fig. 1.

### Clinical parameters

Patient data were obtained from electronic hospital information systems:

Age at diagnosis, gender, histology, tumor localization, histological grading according to FNCLCC (Fédération Nationale des Centres de Lutte Contre le Cancer) for STS [24, 25], TNM classification, tumor size, location of metastases, date of diagnosis, date of histology, date of relapse/progression, location of relapse, date of death, date of surgery, status of resection, chemotherapy regimens and radiation therapies. OSS and CS were graded according to the WHO classification [26]; all ES were graded as G3. TNM staging was done according to UICC (Union Internationale Contre le Cancer), TNM classification for malignant tumors (9th edition) with T classification according to anatomical location for STS and separate TNM staging classification for each BS [27]. Event-free survival (EFS) was calculated as the time to relapse after surgery or time to progression after the first systemic therapy depending on resection status. Overall survival (OS) was calculated as the time from diagnosis to death.

### Statistical analysis

**Fig. 2:**
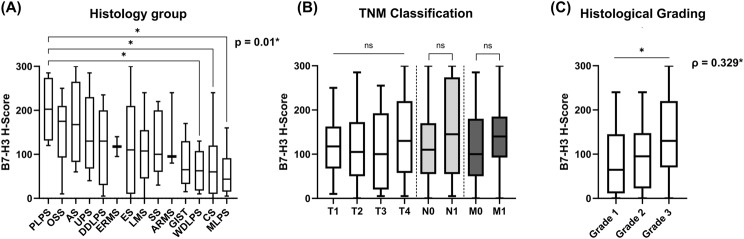
B7-H3 expression correlates with higher histological grading. **A** B7-H3 H-Scores according to sarcoma histology. Kruskal-Wallis test was significant (*p* = 0.01) and PLPS was found to have significantly higher B7-H3 expression than WDLPS, CS and MLPS. **B** B7-H3 H-Scores according to initial TNM classification. **C** Spearman correlation shows a significant correlation between B7-H3 H-Scores and histological grading. Box plots show median, IQR and range. Median H-Scores are shown in Table 2

GraphPad Prism 9.4.1 (Dotmatics, Boston, MA, USA) and SPSS Statistics 30 (IBM, Armonk, NY, USA) were used for statistical analysis. B7-H3 H-Scores were non-normally distributed according to Shapiro-Wilk tests. Therefore, Mann-Whitney-U tests were used for pairwise group comparisons and Kruskal-Wallis tests were employed for multiple group comparisons. Dunn’s post-hoc test was performed for PLPS group comparisons in Fig. 2B. Continuous variables were correlated by Spearman’s rank correlation.

Survival analysis was conducted according to the Kaplan-Meier method. Patients were censored when no EFS or OS event had occurred at the last follow-up. Survival curves were compared by pairwise log-rank tests. Cox proportional hazards regression was conducted for EFS with the covariables sex, age, staging according to TNM, histology (BS or STS) and B7-H3 H-Score. No outliers were removed from the dataset. The proportional hazards assumption was checked for all covariates. Grading was excluded from Cox regression due to collinearity with B7-H3 H-Score. Histology was dichotomized to BS or STS to reduce the variable to event ratio. No Cox regression was performed for OS due to the low number of OS events (*n* = 24, 20%).

Significance level was *p* < 0.05 for all statistical tests and significant comparisons were marked with asterisks. Alpha error correction was conducted according to Bonfferoni.

### Data availability

The datasets generated and analyzed during the current study are not publicly available due to patient data protection and privacy but are available from the corresponding author on reasonable request.

## Results

### Patient cohort

A total of 133 sarcoma patients with available pretherapeutic specimens could be identified in the sarcoma centers of the University Hospitals of Tübingen and Freiburg. Most patients had soft tissue sarcomas (STS, *n* = 103), with the most frequent diagnoses being LPS (26%), UPS (14%) and LMS (11%). 23% of patients (*n* = 30) had bone sarcomas. All histological gradings were represented in the cohort and 20% of patients had distant metastasis at the time of diagnosis (Table 1). Most patients received resection of their primary tumors (87%), 35% had neoadjuvant therapies, 32% received adjuvant therapies and 34% received palliative therapies, mostly containing chemotherapy (Table S1).

**Table 1:** Characteristics of the sarcoma patient cohort

Characteristics	Total patient number (*n* = 133)
Sex	
Male	57% (*n* = 76)
Female	43% (*n* = 57)
Median Age	61 (IQR 43–73)
Histology groups	
Undifferentiated pleomorphic sarcoma (UPS)	14% (*n* = 18)
Leiomyosarcoma (LMS)	12% (*n* = 16)
Myxoid liposarcoma (MLPS)	9% (*n* = 12)
Dedifferentiated liposarcoma (DDLPS)	8% (*n* = 11)
Chondrosarcoma (CS)	8% (*n* = 11)
Synovial Sarcoma (SS)	8% (*n* = 11)
Ewing Sarcoma (ES)	7% (*n* = 9)
Osteosarcoma (OSS)	7% (*n* = 9)
Angiosarcoma (AS)	7% (*n* = 9)
Well-differentiated liposarcoma (WDLPS)	5% (*n* = 6)
Gastrointestinal stromal tumor (GIST)	4% (*n* = 5)
Pleomorphic liposarcoma (PLPS)	3% (*n* = 4)
Alveolar Rhabdomyosarcoma (ARMS)	3% (*n* = 3)
Embryonal Rhabdomyosarcoma (ERMS)	2% (*n* = 2)
Other	9% (*n* = 7)
Histological Grading according to FNCLCC/WHO	
1	15% (*n* = 20)
2	27% (*n* = 36)
3	52% (*n* = 69)
Staging according to TNM	
Tumor	
T1	23% (*n* = 30)
T2	28% (*n* = 38)
T3	17% (*n* = 22)
T4	25% (*n* = 33)
Lymphnodes	
N0	91% (*n* = 121)
N1	8% (*n* = 10)
Metastases	
M0	80% (*n* = 107)
M1	20% (*n* = 26)
Median tumor diameter (cm)	9.4 (IQR 6.7–14.75)
Median B7-H3 H-Score	110 (IQR 55–180)
B7-H3 negative (0)	3% (*n* = 4)
B7-H3 low (1–100)	45% (*n* = 59)
B7-H3 intermediate (101–200)	33% (*n* = 43)
B7-H3 high (201–300)	19% (*n* = 25)
Event-free survival (EFS, relapse or progression)	
Events	54% (*n* = 72)
Local relapse	39% (*n* = 28)
Distant relapse	67% (*n* = 48)
Kaplan-Meier estimate for median EFS	605 d
Median follow-up	1198 d
Overall survival (OS)	
Events	19% (*n* = 25)
Kaplan-Maier estimate for median OS	N/A
Median follow-up	981 d

All CS were of conventional subtype and the OSS subgroup contained eight conventional OSS (either osteoblastic or chondroblastic), of which one was a secondary OSS. Histological subtypes summarized under “other“ were gastrointestinal stromal tumor (GIST), epithelioid sarcoma, chordoma, giant cell tumor of the bone (GCTOB), chordoma, myxofibrosarcoma (MFS), alveolar soft part sarcoma (ASPS) and malignant peripheral nerve sheath tumor (MPNST)

### B7-H3 surface expression across sarcoma subtypes

B7-H3 H-Scores were divided in four different categories, with four patients (3%) staining completely negative for B7-H3, two of them with Ewing Sarcomas. H-Scores ranging from 1 to 100 were classified as “low” (59 patients, 45%), ranging from 101 to 200 as intermediate (43 patients, 33%) and ranging from 201 to 300 as “high” (25 patients, 19%, Fig. 1). Highest median H-Scores were found in PLPS (Median 202.5, IQR 131–274), OSS (Median 175, IQR 92.5–210) and AS (median 167.5, IQR 82.5–265), the lowest H-Scores had patients with MLPS, (median 44.5, ICR 15–91), CS (median 60, IQR 10–120) and WDLPS (median 62.5, IQR 17.5–107.5). Large variances in H-Scores were found between individual samples of the same histologies (Fig. 2A), reflected by large IQRs. B7-H3 surface expression of PLPS was found to be significantly higher than in MLPS (*p* = 0.01), CS (*p* = 0.034) and WDLPS (*p* = 0.038). Initial tumor size (T status), nodal status or metastases at baseline staging did not correlate with B7-H3 H-Scores (Fig. 2B). The H-Scores found in very rare sarcoma subtypes with low samples sizes are shown separately (Table S2). 

### Histological grading correlates with B7-H3 expression

B7-H3 H-Scores were found to be significantly higher in sarcomas with higher histological grading (Fig. 2C). For STS, FNCLCC grading was employed, whereas grading according to WHO was employed for BS. ES were considered G3 (high-grade). The spearman correlation coefficient was 0.329 (95% CI 0.156–0.482, *p* < 0.001), demonstrating a significant positive correlation between higher histological grading and B7-H3 H-Scores throughout all sarcoma subtypes (Table 2; Fig. 2C). In the histological groups of STS, B7-H3 expression also significantly correlated with grading. In the BS subgroup, no significant correlation was found, albeit with low sample numbers of only *n* = 3 in the BS G2 subgroup (Fig. S1). B7-H3 H-Scores did not differ significantly between sex, histological sarcoma subtypes, nodal status or initial metastases. Further, no correlation between B7-H3 H-Score and age or T status was found (Table 2).

**Table 2:** Baseline parameters and B7-H3 H-Scores

Characteristics	Patient number (*n* = 133)	Median H-Score	*P*-value (Mann-Whitney-U or Kruskal-Wallis Test)	Correlation coefficient (Spearman)
Sex			0.38	
Male	76	100		
Female	57	120		
Age	133	110		0.098
Sarcoma subtype			0.939	
Soft tissue sarcoma	103	105		
Bone sarcoma	30	110		
Histology group			0.01*	
Pleomorphic liposarcoma (PLPS)	4	202.5		
Other	7	180		
Osteosarcoma (OSS)	8	175		
Angiosarcoma (AS)	8	167.5		
Undifferentiated pleomorphic sarcoma (UPS)	18	130		
Dedifferentiated liposarcoma (DDLPS)	11	130		
Embryonal Rhabdomyosarcoma (ERMS)	2	117.5		
Ewing Sarcoma (ES)	9	110		
Leiomyosarcoma (LMS)	16	107.5		
Synovial Sarcoma (SS)	11	100		
Alveolar Rhabdomyosarcoma (ARMS)	3	95		
Gastrointestinal stromal tumor (GIST)	5	65		
Well-differentiated liposarcoma (WDLPS)	6	62.5		
Chondrosarcoma (CS)	11	60		
Myxoid liposarcoma (MLPS)	11	44.5		
Histological Grading according to FNCLCC/WHO				0.329*
1	20	64.5		
2	36	95		
3	67	130		
Staging according to TNM				
Tumor				0.02
T1	30	117.5		
T2	38	105		
T3	22	100		
T4	33	130		
Lymphnodes			0.25	
N0	113	110		
N1	10	145		
Metastases			0.144	
M0	99	100		
M1	24	145		

A Kruskal-Wallis test showed a significant difference in B7-H3 H-Scores between the sarcoma histology groups (*p* = 0.01). Spearman Correlation between grading and H-Score was also significant with a correlation coefficient of 0.329 (95% CI 0.156–0.482, *p* < 0.001)

*p<  0.05

### High and intermediate B7-H3 H-Scores are associated with shorter event free survival

**Fig. 3:**
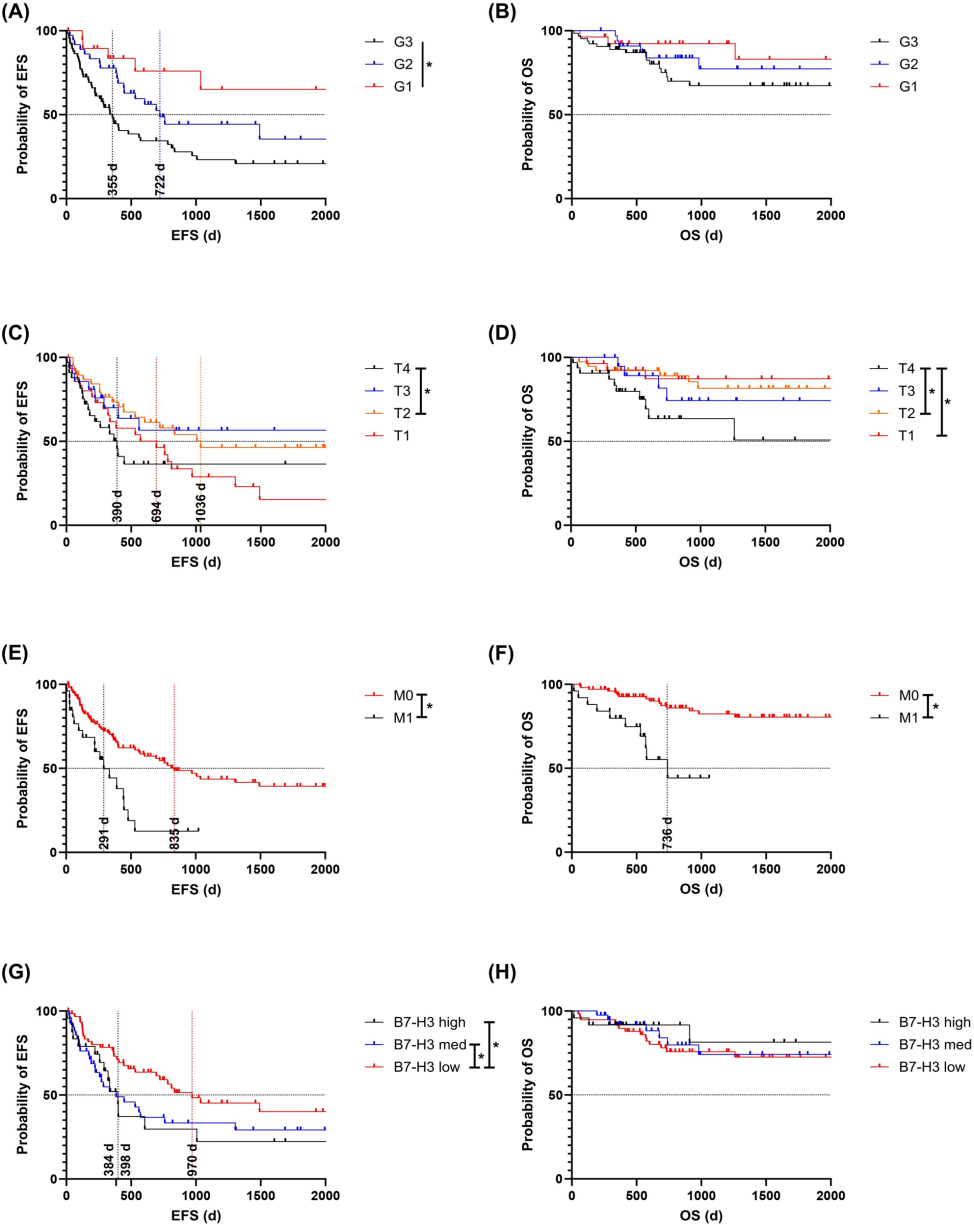
EFS and OS of the sarcoma cohort. Kaplan-Mayer survival curves for EFS (left column) and OS (right column) according to histological grading (**A** & **B**), T stadium (**C** & **D**), initial metastases (**E** & **F**) and B7-H3 H-Score groups (**G** & **H**). Vertical lines indicate Kaplan-Mayer estimates for median EFS and OS. Vertical ticks indicate censoring of patients. Paired Log Rank tests were conducted and asterisks indicate *p* < 0.05

During a median follow-up period of approximately 3 years, 72 patients experienced relapse or disease progression and 25 patients died (Table 1). Median EFS was 605 days and median OS was not reached. Higher histological grading was associated with significantly shorter EFS (Fig. 3A, median EFS G3 355 days vs. G2 722 d vs. G1 not reached, *p* = 0.004). Higher histological grading showed a trend toward an increased hazard radio (HR) for death (Fig. 3B), but without statistical significance. Also, a higher T status was associated with shorter EFS (Fig. 3C, median EFS T4 390 d vs. T1 694 d vs. T2 not reached, T4 vs. T2, *p* = 0.042). Patients with T4 sarcomas had significantly shorter OS compared to T1 or T2 sarcomas (Fig. 3D, median OS not reached T4 vs. T1, *p* = 0.018; T4 vs. T2, *p* = 0.036). Metastases at baseline were also a significant predictor for shorter EFS (Fig. 3E, median EFS 291 d vs. 835 d, *p* < 0.001) and OS (Fig. 3F, median OS 736 d vs. not reached, *p* < 0.001). The B7-H3 H-Score group also correlated with EFS estimates, with a median EFS of 384 d for B7-H3 high, 398 d for B7-H3 intermediate and 970 d for B7-H3 low expressing sarcomas (Fig. 3G). High or intermediate B7-H3 H-Score were significantly associated with shorter EFS compared to the B7-H3 low group (Fig. 3G, B7-H3 high vs. B7-H3 low *p* = 0.044, B7-H3 intermediate vs. B7-H3 low *p* = 0.043). For OS, no differences were found between the three B7-H3 H-Score groups (Fig. 3H). No significant differences were found between B7-H3 H-Scores and the overall number of EFS or OS events and the location of progression (Table S3).

### B7-H3 H-Score predicts EFS across sarcoma subtypes

**Fig. 4:**
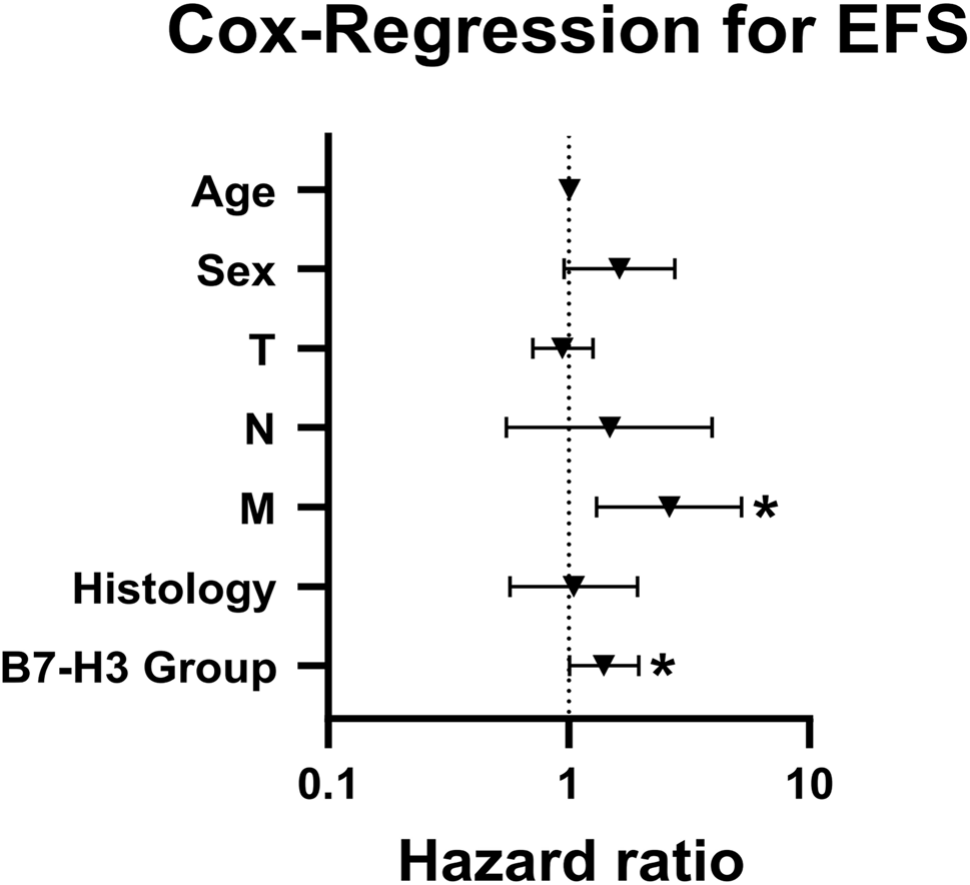
Cox proportional hazards regression model for EFS. Age, sex, TNM Staging, histology (Bone Sarcoma (BS) vs. Soft Tissue Sarcoma (STS)) and B7-H3 H-Score group predict EFS in a Cox proportional hazards regression model. Histological grading was excluded because of collinearity with B7-H3 H-Score. Forrest plot shows hazard ratios (HRs) and 95% confidence intervals (CIs) for EFS. Asterisks indicate *p* < 0.05

A multivariable Cox proportional hazards regression model was employed to estimate the association of B7-H3 H-Score group and EFS and adjust for age (continuous), sex (male vs. female), T status (continuous), nodal status (N0 vs. N1), initial metastases (M0 vs. M1) and sarcoma histology (BS vs. STS) (Fig. 4). Histological grading was removed from the model because of high collinearity with B7-H3 H-Scores. An omnibus test for the global null hypothesis was performed using the likelihood ratio test. The test statistic was X^2^ = 18.8 with 7 degrees of freedom (*p* = 0.009), indicating that the model is statistically significant. B7-H3 H-Score was found to be a significant independent predictor for shorter EFS (HR 1.4, 95% CI 1.01–1.99, *p* = 0.044). Further, initial metastases were a significant independent predictor for shorter EFS (HR 2.62, 95% CI 1.32–5.22, *p* = 0.006). Age, sex, T stadium and nodal status did not independently predict EFS (Fig. 4).

## Discussion

Despite innovative approaches, sarcomas still lack promising treatment options, especially in surgically uncurable and metastatic disease. B7-H3 is a promising novel treatment target with a broad pipeline of B7-H3 directed treatment strategies under investigation in a variety of tumor entities. High target expression on the cellular surface is a prerequisite for successful implementation of such therapies [28].

To our knowledge, this is the first study with a pan-sarcoma cohort using only pre-treatment samples from primary tumors. B7-H3 H-Scores from IHC were chosen to reflect the B7-H3 tumor cell surface expression and intratumoral heterogeneity of B7-H3 expression in sarcomas. Here, 97% of sarcomas showed at least low expression of B7-H3 on the cellular surface. The overall median B7-H3 H-Score was 110 and the subtypes PLPS, OSS and AS had higher B7-H3 expression, whereas WDLPS, CS and MLPS showed lower tumor cell surface expression. Statistical significance was reached for B7-H3 expression in PLPS when compared to the other LPS subtypes MLPS and WDLPS as well as CS. Overall, H-Score variances within the same histology were high.

B7-H3 surface expression was significantly correlated with histological grading in the whole sarcoma cohort as well as in the STS cohort. Other analyses have showed comparable B7-H3 expression in sarcomas. In the largest analysis of B7-H3 expression in STS to date, a cohort of 153 specimens representing 15 different STS subtypes, both before and after therapy, B7-H3 was assessed using an immunochemistry (IHC) score from zero to five. B7-H3 was found to be highly expressed in 69% of specimens and 97% of samples showed at least low expression. No correlation was found between PD-L1 expression, grading, prior treatment, tumor size or age and influence of B7-H3 expression on progression free survival (PFS) was not studied. However, samples during and post-treatment as well as from metastatic lesions were included in this analysis, which both might influence B7-H3 surface expression [22]. In RMS, B7-H3 was already found to be highly upregulated compared to normal muscle tissue and B7-H3 expression was associated with reduced CD8^+^ cell infiltration and identified as a mechanism for tumor immune evasion. Median H-Scores were found to be 70 in 132 embryonal and alveolar RMS specimens, but with a major intratumoral heterogeneity [20]. For OSS, B7-H3 was found to be overexpressed in the vast majority of the samples and correlated with faster recurrence, short overall survival and reduced CD8^+^ cell infiltration [29, 30]. A collection of samples from pediatric malignancies revealed positivity from 89 to 100% for pediatric ARMS and ES with approximately one third of samples with the highest staining intensity (3+). In the same study, B7-H3 targeted CAR-T-Cells showed promising efficacy against ES and OS xenografts in mice with high dependance on surface target antigen density [28].

In this study, membranous B7-H3 expression could be identified as a predictor for EFS but not OS. The B7-H3 group analysis dividing low, intermediate and high B7-H3 expression showed shorter EFS with higher B7-H3 expression. After correction for sex, age, histology and TNM classification using Cox regression, membranous B7-H3 expression was found to be an independent predictor for EFS. This effect could also be explained by the significant correlation with histological grading which was also found to be associated with EFS and survival outcomes. As a limitation, histological grading was therefore be excluded from the Cox regression model because of collinearity. Metastases at baseline were another independent predictor for EFS, validating this patient cohort. Preclinical sarcomas models link B7-H3 expression to invasiveness, cell migration and blocked cellular differentiation, potentially indicating a more aggressive phenotype in B7-H3 high expressing sarcomas [20, 31]. A previous study found B7-H3 expression on a transcriptomic level as a prognostic parameter in bone sarcomas, but without correcting for potential confounders [15]. Holzmayer et al. used the TCGA (The Cancer Genome Atlas) database and correlated B7-H3 expression data of 231 sarcoma transcriptomes with PFS and OS. B7-H3 expression in the highest quartile significantly correlated with poorer OS and EFS compared to the lowest quartile [19]. Compared to this study, transcriptomic data were used and no correction for confounding variables was conducted in both previous studies.

Differences to previous transcriptomic studies can be explained by (i) using only pre-treatment samples which (ii) were all derived from primary tumors and by (iii) a different readout using IHC for surface expression. Bulk-RNA sequencing for B7-H3 comes with limitations, especially for sarcomas. Bulk transcriptome data do not resolve tumor subclones, stromal and immune content, or spatial heterogeneity of B7-H3 expression [15]. Further, B7-H3 is highly expressed on an RNA level in several benign tissues but downregulated on a posttranscriptional level [8]. Therefore, IHC data remain the gold standard for tumor surface expression of this therapy target.

Regarding OS, only 25 deaths (19%) were recorded in this cohort. Most patients (80%) had localized disease and received curative treatment. Therefore, conclusions on B7-H3 H-Score and survival were limited in this study. Shorter EFS as well as the correlation with histological grading indicate more aggressive tumor biology and are both associated with high B7-H3 surface expression. That may translate into poorer OS outcomes after a longer observation period and a higher number of OS events. In other tumor entities such as prostate cancer, cervical cancer or renal carcinoma, high B7-H3 expression was correlated with adverse survival prognosis and more aggressive disease [32–34].

As another limitation, no post-treatment samples and no follow-up data on B7-H3 surface expression were available in this study. Previous IHC data from non-small cell lung cancer (NSCLC) suggest that chemotherapy or radiation can alter B7-H3 expression through the disease course [35].

To date, only few results from clinical studies using B7-H3 targeted therapeutics in sarcoma have been reported. The Fc optimized monoclonal antibody Enoblituzumab (MGA271) was employed in a pediatric clinical trial including six patients with OSS, six patients with ES and two patients with RMS. The antibody was found to be safe and induced inflammatory responses in some patients, but no objective responses were noted [17]. In the ARTEMIS-002 trial (NCT05830123), the B7-H3 targeted antibody drug conjugate (ADC) HS-20093 is evaluated in relapsed and refractory sarcomas. Interim results demonstrated an objective response rate (ORR) of 20% for 21 patients with osteosarcoma, albeit with a short follow-up time (4.1 months) [36]. Recently, a trial update confirmed an ORR of 20% in 45 osteosarcoma patients, with a promising median PFS of 8.4 months. In 13 STS patients, ORR was 23.1% and median PFS 9.4 months, demonstrating clinical activity of this B7-H3 targeted ADC. The drug is currently assessed for combinatorial treatments in advanced BS and STS in another Phase Ib study (NCT06699576). More clinical trials targeting B7-H3 in solid tumors or specifically in sarcomas encompassing other ADCs (NCT06242470), cell therapies (NCT06500819, NCT04897321) or bsABs (NCT05999396 [18]) are recruiting.

In conclusion, this comprehensive study clarifies the landscape of B7-H3 surface expression as a therapeutic target in sarcomas. Particularly high-grade sarcomas, PLPS, OSS and AS show high surface expression, making them promising tumor entities for B7-H3 instructed therapy. Further, B7-H3 surface expression was associated with faster relapse and tumor progression. Patients with relapsed or refractory sarcomas still have a poor prognosis and few conventional therapy options and should therefore be referred to suitable study treatments. B7-H3 testing should also be incorporated in therapeutic clinical trials for biomarker identification.

## Supplementary Information

Supplementary Material 1.

## Data Availability

Additional data can be found in the supplementary material. The datasets generated and analyzed during the current study are not publicly available due to patient data protection and privacy but are available from the corresponding author on reasonable request.

## Abbreviations

ADC: Antibody drug conjugate
ARMS: Alveolar rhabdomyosarcoma
AS: Angiosarcoma
ASPS: Alveolar soft part sarcoma
AZ: Arizona
B7-H3: B7 Homolog 3
BS: Bone sarcoma
bsAb: Bispecific antibody
CA: California
CAR: Chimeric antigen receptor
CD: Cluster of differentiation
CI: Confidence interval
CS: Chondrosarcoma
DDLPS: Dedifferentiated liposarcoma
EDTA: Ethylenediaminetetraacetic acid
EFS: Event-free survival
EMA: European Medicines Agency
ERMS: Embryonal rhabdomyosarcoma
ES: Ewing sarcoma
FDA: Food and Drug Administration
FNCLCC: Fédération Nationale des Centres de Lutte Contre le Cancer
GCTOB: Giant cell tumor of the bone
GIST: Gastrointestinal stromal tumor
H&E: Hematoxylin and Eosin
HR: Hazard ratio
IHC: Immunohistochemistry
IQR: Interquartile range
IRB: Institutional Review Board
LMS: Leiomyosarcoma
LPS: Liposarcoma
MA: Massachusetts
mAB: Monocloncal antibody
MFS: Myxofibrosarcoma
MLPS: Myxoid liposarcoma
MPNST: Malignant peripheral nerve sheath tumor
miRNA: Micro RNA
NK-cell: Natural killer cell
NSCLC: Non-small cell lung cancer
NY: New York
ORR: Objective response rate
OS: Overall survival
OSS: Osteosarcoma
PD-L1: Programmed death-ligand 1
PFS: Progression free survival
PLPS: Pleomorphic liposarcoma
RMS: Rhabdomyosarcoma
SS: Synovial sarcoma
STS: Soft tissue sarcoma
TCGA: The Cancer Genome Atlas
TRIS: Tris(hydroxymethyl)aminomethan
UPS: Undifferentiated pleomorphic sarcoma
USA: United States of America
WDLPS: Well-differentiated liposarcoma
WHO: World Health Organization
