# Supplementary material for: B7-H3 membranous expression correlates with histological grading in a two-center cohort of 133 pre-treatment bone and soft tissue sarcoma samples
Source: BMC Cancer. 2026 Jun 6;26:740. doi: 10.1186/s12885-026-16255-0 (PMC13251000; doi:10.1186/s12885-026-16255-0)
Supplement: Supplementary file 1 — Supplementary Material 1. [file 12885_2026_16255_MOESM1_ESM.docx]

**Supplementary Material:**


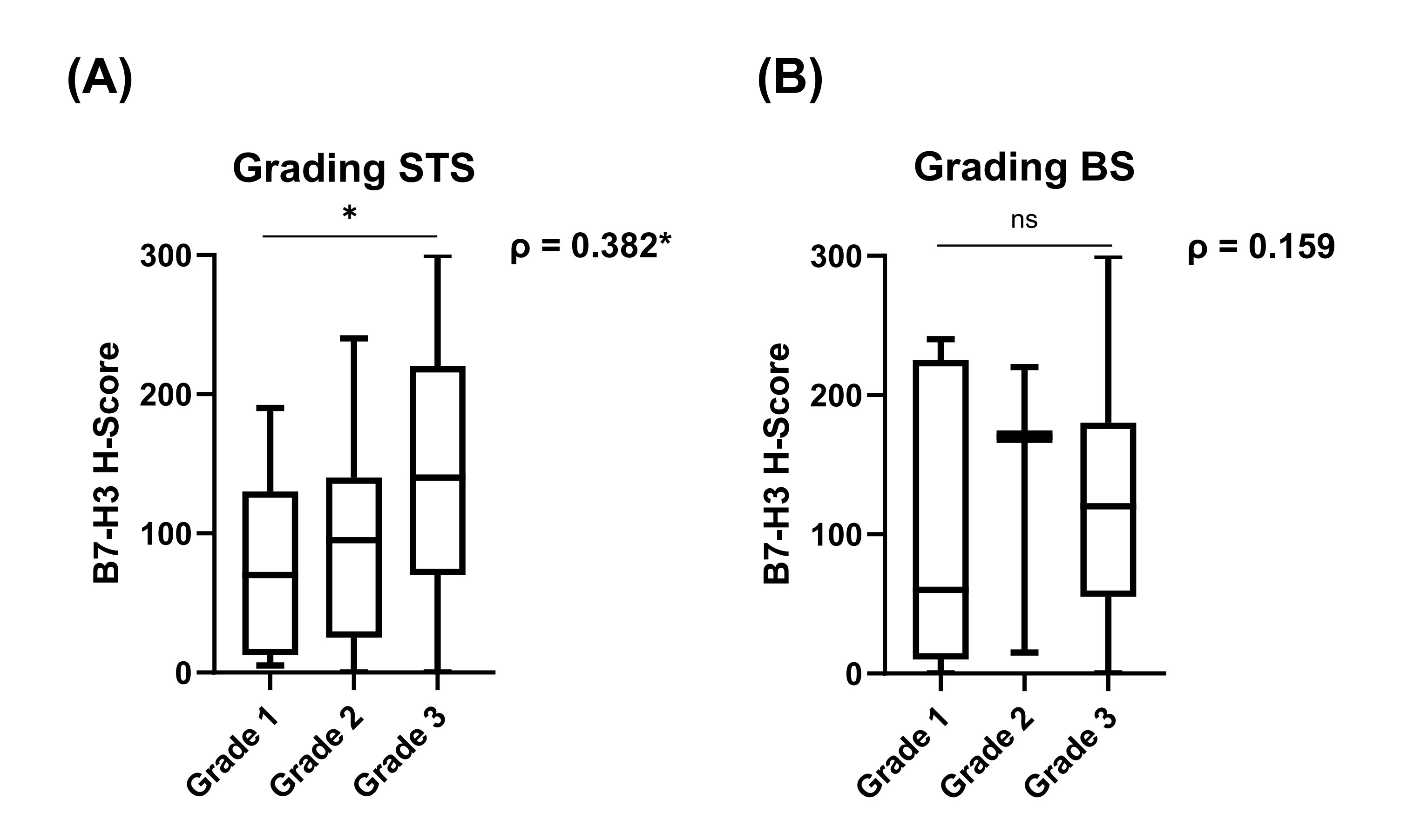


**Figure S1: B7-H3 H-Scores and grading in STS and BS subgroups**

(A) B7-H3 H-Score significantly correlates with grading in the STS subgroup of the sarcoma cohort (n = 104) with median H-Scores of 70 for G1, 95 for G2 and 140 for G3 STS. Spearman correlation coefficient 0.382 (95 % CI 0.19 – 0.546, p < 0.001).

(B) B7-H3 H-Scores according to grading in the BS subgroup (n = 33). Median H-Scores were 60 for G1, 170 for G2 and 120 for G3, however with only n = 3 samples in the G2 BS subgroup. Spearman correlation coefficient 0.159 (95 % CI -0.238 – 0.511, ns). Box plots show median, IQR and range.

| **Treatment modality** | **Total patient number (n = 133)** |
| --- | --- |
| **Neoadjuvant therapy**  **Chemotherapy**  **Radiation**  **Hyperthermia** | 35 % (n = 46)  33 % (n = 44)  18 % (n = 24)  14 % (n = 19) |
| **Resection of primary tumor**  **Initial resection**  **R0**  **R1/R2**  **After neoadjuvant Therapy**  **R0**  **R1/R2**  **No Resection** | 87 % (n = 116)  57 % (n = 76)  74 % (n = 56)  26 % (n = 20)  30 % (n = 40)  83 % (n = 33)  17 % (n = 7)  13 % (n = 17) |
| **Adjuvant therapy**  **Chemotherapy**  **Radiation** | 32 % (n = 43)  20 % (n = 27)  12 % (n= 16) |
| **Palliative therapy**  **Chemotherapy**  **Radiation**  **Surgery** | 34 % (n = 45)  26 % (n = 37)  13 % (n = 17)  5 % (n = 7) |

**Table S1: Treatment modalities in the sarcoma cohort.**

| **Characteristics** | **Patient number (n = 12)** | **Median**  **H-Score** | **Grading according to FNCLCC** |
| --- | --- | --- | --- |
| **Histology**  **GCTOB**  **Epithelioid sarcoma**  **Chordoma**  **FMS**  **ASPS**  **MPNST** | **1**  **1**  **1**  **2**  **1**  **1** | **240**  **160**  **180**  **175**  **140**  **180** | **N/A**  **N/A**  **N/A**  **1**  **2**  **3** |

**Table S2: B7-H3 H-Scores found in rare histologies summarized under “other” in Table 1. GCTOB: giant cell tumor of the bone, FMS: fibromyxosarcoma, ASPS: alveolar soft part sarcoma, MPNST: malignant peripheral nerve sheath tumor.**

| **Characteristics** | **Patient number (n = 133)** | **Median**  **H-Score** | **p-value (Mann-Whitney-U Test)** |
| --- | --- | --- | --- |
| **Relapse or progression**  **No**  **Yes** | **58**  **71** | **92.5**  **115** | **0.297** |
| **Location of progression**  **Local**  **Distant** | **24**  **47** | **125**  **110** | **0.451** |
| **Death**  **No**  **Yes** | **99**  **24** | **120**  **97.5** | **0.496** |

**Table S3: Outcome parameters and B7-H3 H-Scores.**
